# Supplementary material for: Characterization of terminal flowering cowpea (Vigna unguiculata (L.) Walp.) mutants obtained by induced mutagenesis digs out the loss-of-function of phosphatidylethanolamine-binding protein
Source: PLoS One. 2023 Dec 14;18(12):e0295509. doi: 10.1371/journal.pone.0295509 (PMC10721064; doi:10.1371/journal.pone.0295509)
Supplement: S1 Fig — (DOCX) [file pone.0295509.s001.docx]

***
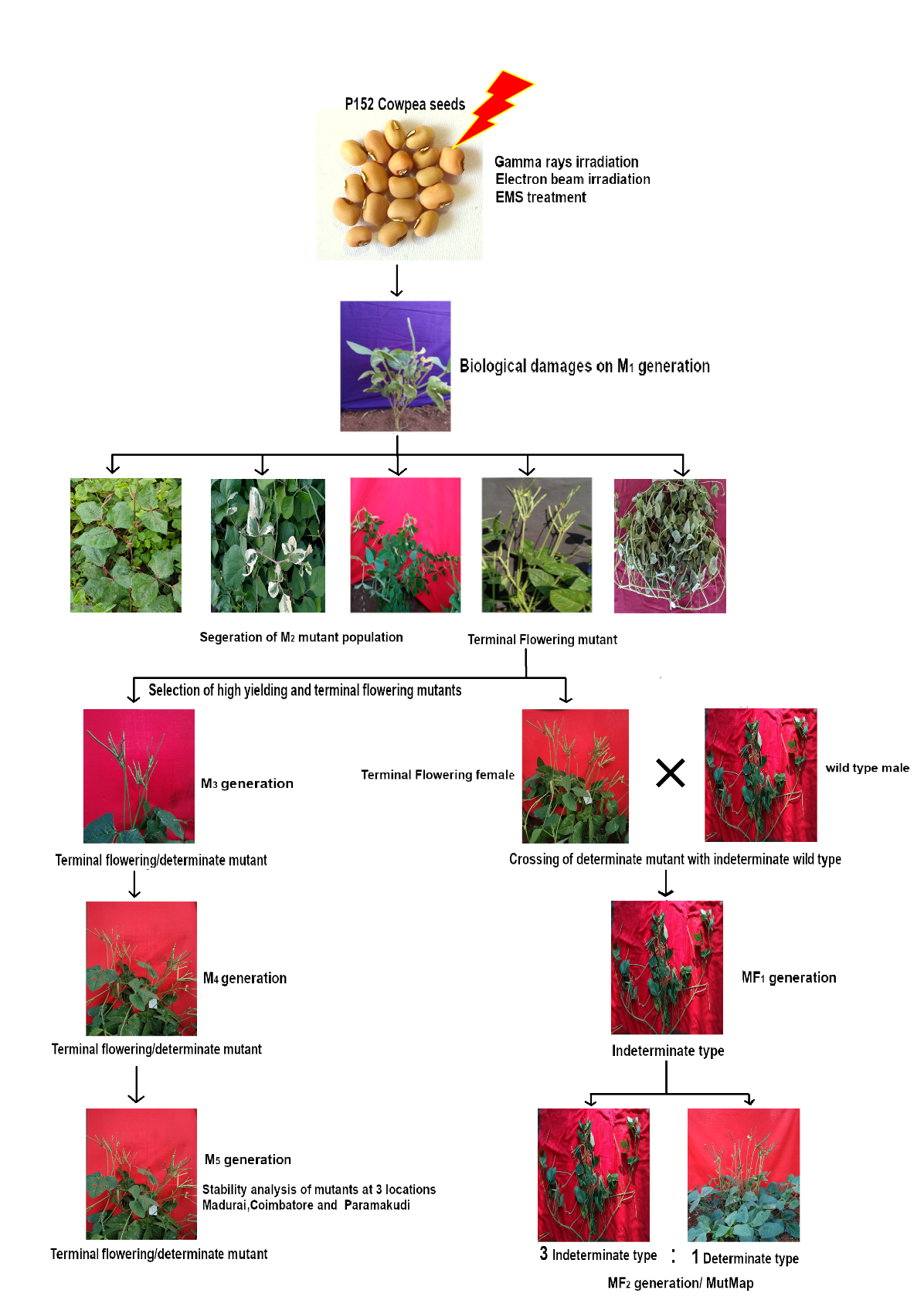
***

**S1 Fig. Schematic representation for development of terminal flowering mutants and MutMap population in cowpea.**
