# Supplementary figures and images for: Characterization of terminal flowering cowpea (Vigna unguiculata (L.) Walp.) mutants obtained by induced mutagenesis digs out the loss-of-function of phosphatidylethanolamine-binding protein
Source: PLoS One. 2023 Dec 14;18(12):e0295509. doi: 10.1371/journal.pone.0295509 (PMC10721064; doi:10.1371/journal.pone.0295509)

**
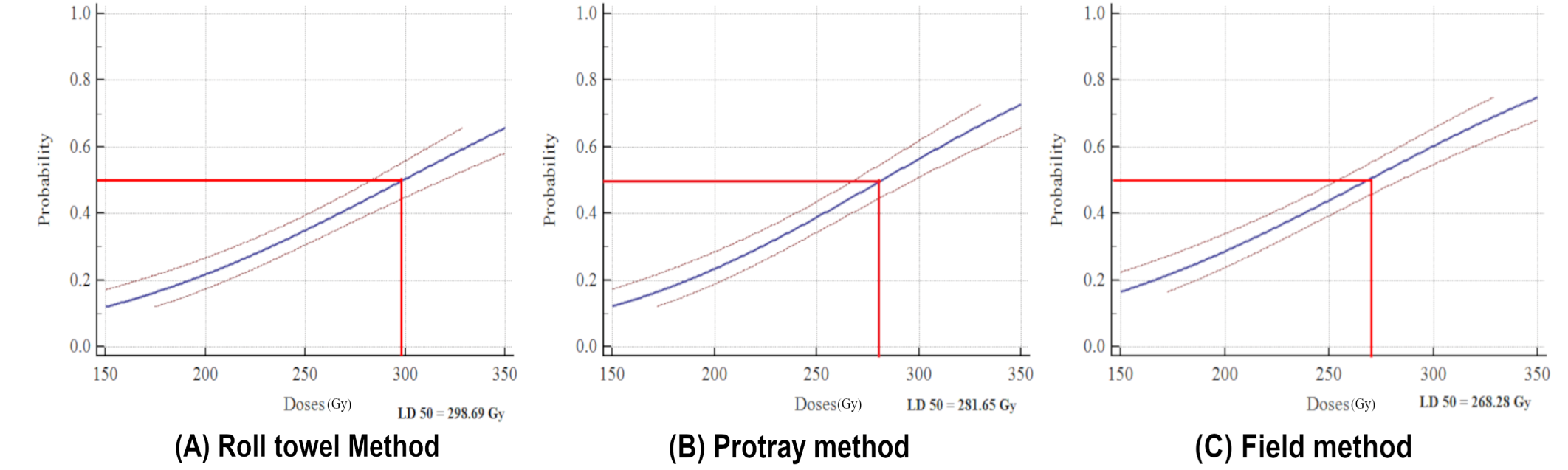
S2 Fig. Mutagenic dosage optimization of gamma rays in P152 cowpea cultivar through LD50.**

Supplement: S2 Fig — (DOCX) [file pone.0295509.s002.docx]

**
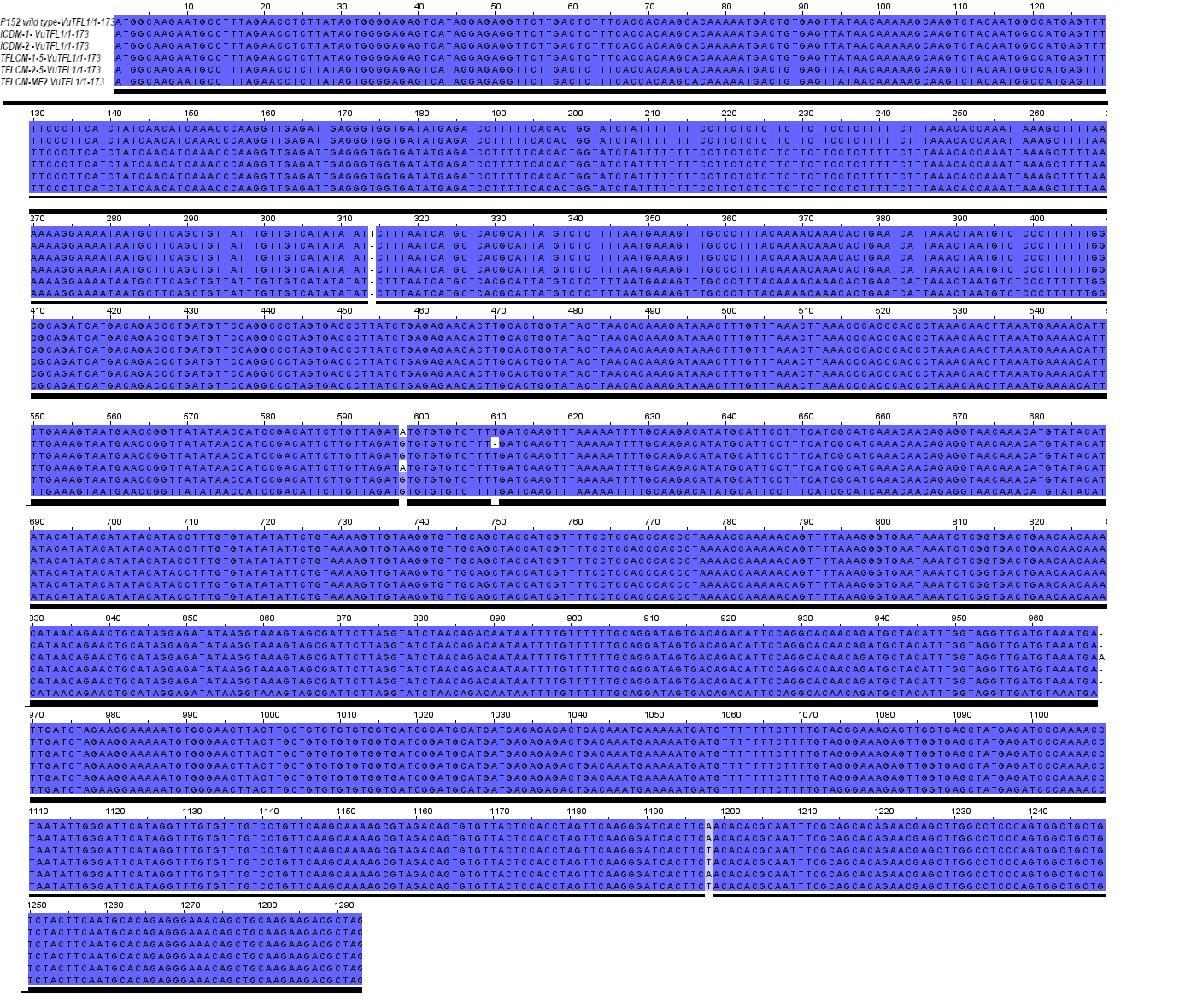
**

**S4 Fig. Multiple sequence alignment of *VuTFL1* gene in cowpea mutants.**

Supplement: S4 Fig — (DOCX) [file pone.0295509.s004.docx]
