# Supplementary material for: Characterization of terminal flowering cowpea (Vigna unguiculata (L.) Walp.) mutants obtained by induced mutagenesis digs out the loss-of-function of phosphatidylethanolamine-binding protein
Source: PLoS One. 2023 Dec 14;18(12):e0295509. doi: 10.1371/journal.pone.0295509 (PMC10721064; doi:10.1371/journal.pone.0295509)
Supplement: S3 Fig — (DOCX) [file pone.0295509.s003.docx]

**
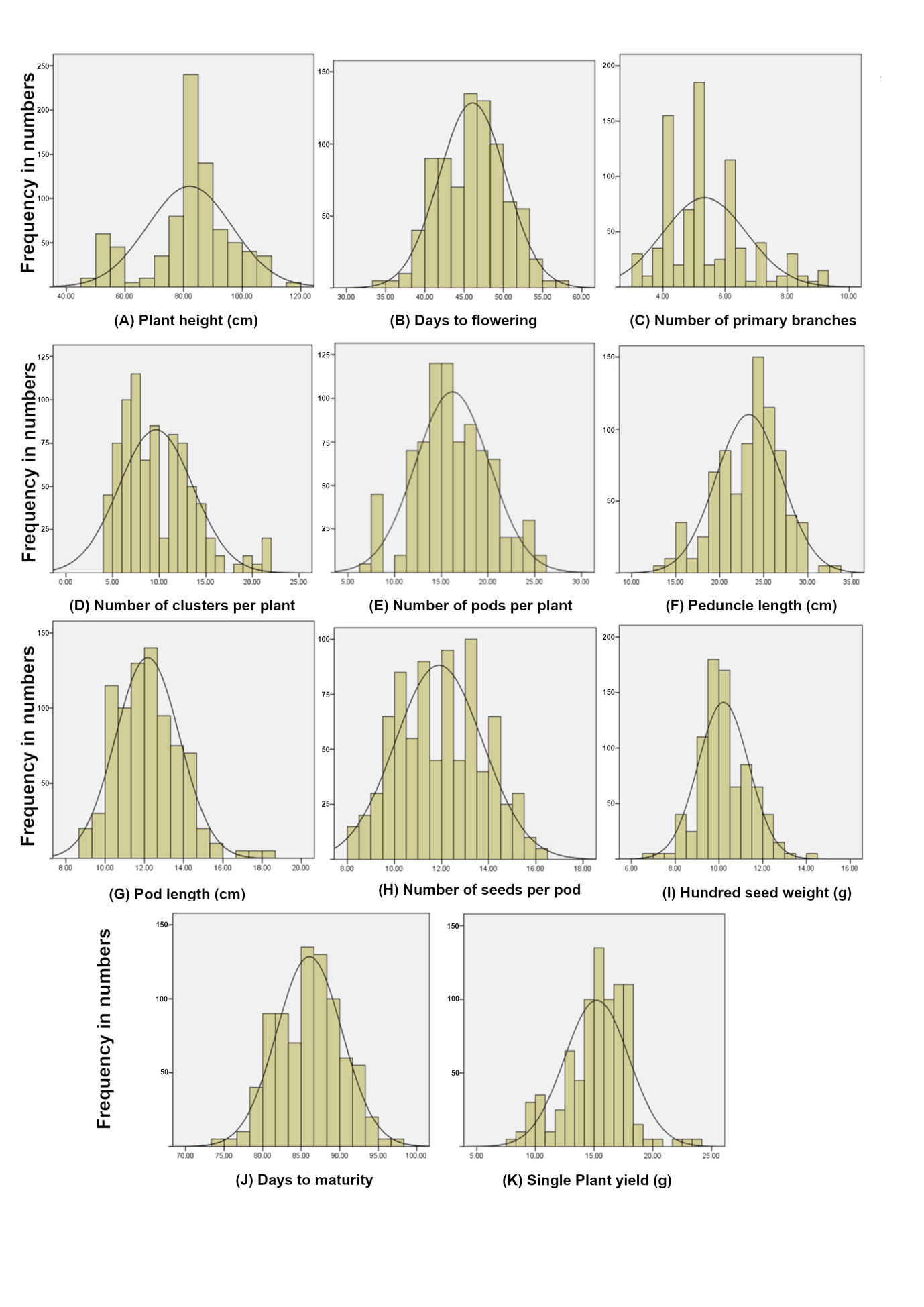
S3 Fig. Frequency distribution of various morphological traits in M_3_ generation of cowpea cultivar P152 generated by gamma irradiation.**
