# Supplementary material for: Characterization of terminal flowering cowpea (Vigna unguiculata (L.) Walp.) mutants obtained by induced mutagenesis digs out the loss-of-function of phosphatidylethanolamine-binding protein
Source: PLoS One. 2023 Dec 14;18(12):e0295509. doi: 10.1371/journal.pone.0295509 (PMC10721064; doi:10.1371/journal.pone.0295509)
Supplement: S1 Table — (DOCX) [file pone.0295509.s005.docx]

**S1 Table. Mean, variability and heritability estimates of quantitative traits in M_3_ generation of cowpea cultivar P152 generated by gamma irradiation**

| **S. No.** | **Traits** | **Mutagen dose** | **P 152** | | | | | |
| --- | --- | --- | --- | --- | --- | --- | --- | --- |
|  |  |  | **Mean ± SE** | **Range** | **PCV %** | **GCV %** | **H^2^ %** | **GA %** |
| **1.** | **Plant height (cm)** | **Wild type** | **73.63 ± 0.58** | | | | | |
|  |  | **200 Gy** | 78.23 ± 1.12 | 61.70 – 96.50 | 9.48 | 8.45 | 79.47 | 15.52 |
|  |  | **250 Gy** | 77.45 ± 1.45 | 60.30 – 121.10 | 11.93 | 11.11 | 86.76 | 21.32 |
|  |  | **300 Gy** | 79.85 ± 1.54 | 52.40 – 115.30 | 10.27 | 9.37 | 83.20 | 17.60 |
| **2.** | **Days to flowering** | **Wild type** | **49.54 ± 0.18** | | | | | |
|  |  | **200 Gy** | 52.25 ± 0.54 | 41.0 – 56.0 | 8.10 | 7.23 | 79.86 | 13.32 |
|  |  | **250 Gy** | 51.47 ± 0.61 | 46.0 – 60.0 | 6.27 | 5.07 | 65.39 | 8.44 |
|  |  | **300 Gy** | 54.12 ± 0.82 | 48.0 – 61.0 | 6.83 | 5.86 | 73.61 | 10.35 |
| **3.** | **No. of primary branches** | **Wild type** | **4.08 ± 0.04** | | | | | |
|  |  | **200 Gy** | 4.82 ± 0.21 | 3.0 – 6.0 | 19.65 | 7.97 | 16.45 | 6.66 |
|  |  | **250 Gy** | 5.21 ± 0.18 | 4.0 – 7.0 | 19.94 | 11.07 | 30.83 | 12.66 |
|  |  | **300 Gy** | 5.43 ± 0.16 | 4.0 – 7.0 | 20.29 | 12.51 | 38.03 | 15.89 |
| **4.** | **No. of clusters per plant** | **Wild type** | **10.15 ± 0.07** | | | | | |
|  |  | **200 Gy** | 9.78 ± 0.13 | 5.0 – 13.0 | 15.54 | 9.35 | 36.17 | 11.58 |
|  |  | **250 Gy** | 9.12 ± 0.15 | 6.0 – 15.0 | 19.06 | 13.64 | 51.20 | 20.10 |
|  |  | **300 Gy** | 8.12 ± 0.16 | 4.0 – 16.0 | 18.88 | 11.52 | 37.26 | 14.49 |
| **5.** | **No. of pods per plant** | **Wild type** | **19.08 ± 0.22** | | | | | |
|  |  | **200 Gy** | 17.8 ± 0. 65 | 11.0 – 30.0 | 18.26 | 13.74 | 56.66 | 21.31 |
|  |  | **250 Gy** | 16.42 ± 0.46 | 13.0 – 32.0 | 15.57 | 8.53 | 30.02 | 9.63 |
|  |  | **300 Gy** | 15.4 ± 0.78 | 11.0 – 27.0 | 22.82 | 18.10 | 62.94 | 29.59 |
| **6.** | **Peduncle length (cm)** | **Wild type** | **19.14 ± 0.35** | | | | | |
|  |  | **200 Gy** | 18.63 ± 0.84 | 15.90 – 28.50 | 23.59 | 21.43 | 82.50 | 40.10 |
|  |  | **250 Gy** | 20.45 ± 0.76 | 14.40 – 26.50 | 21.48 | 19.51 | 82.48 | 36.50 |
|  |  | **300 Gy** | 19.63 ± 0.63 | 15.30 – 25.60 | 19.22 | 16.78 | 76.24 | 30.18 |
| **7.** | **Pod length (cm)** | **Wild type** | **11.71 ± 0.11** | | | | | |
|  |  | **200 Gy** | 10.52 ± 0.45 | 8.31 – 14.72 | 22.45 | 15.60 | 48.30 | 22.34 |
|  |  | **250 Gy** | 10.23 ± 0.32 | 7.56 – 14.24 | 22.90 | 15.78 | 47.45 | 22.39 |
|  |  | **300 Gy** | 9.48 ± 0.65 | 7.65 – 14.43 | 30.06 | 24.14 | 64.48 | 39.94 |
| **8.** | **No. of seeds per pod** | **Wild type** | **10.31 ± 0.09** | | | | | |
|  |  | **200 Gy** | 9.24 ± 0.31 | 8.0 – 12.0 | 22.21 | 17.60 | 62.85 | 28.75 |
|  |  | **250 Gy** | 9.19 ± 0.23 | 7.0 – 13.0 | 22.62 | 18.06 | 63.79 | 29.72 |
|  |  | **300 Gy** | 8.54 ± 0.22 | 7.0 – 12.0 | 18.35 | 11.06 | 36.32 | 13.73 |
| **9.** | **Hundred seed weight (g)** | **Wild type** | **9.34±0.03** | | | | | |
|  |  | **200 Gy** | 8.78 ± 0.11 | 7.02 – 10.48 | 11.28 | 8.80 | 60.92 | 14.15 |
|  |  | **250 Gy** | 9.02 ± 0.06 | 7.45 – 15.23 | 11.27 | 8.95 | 62.97 | 14.62 |
|  |  | **300 Gy** | 9.45 ± 0.14 | 7.36 – 10.89 | 10.51 | 8.22 | 61.20 | 13.25 |
| **10.** | **Single plant yield (g)** | **Wild type** | **16.67±0.21** | | | | | |
|  |  | **200 Gy** | 15.23 ± 0.80 | 10.32 – 19.48 | 19.19 | 17.73 | 85.37 | 33.75 |
|  |  | **250 Gy** | 14.23 ± 0.63 | 9.48 – 20.34 | 19.07 | 17.38 | 83.04 | 32.62 |
|  |  | **300 Gy** | 12.13 ± 0.54 | 8.79 – 19.56 | 28.57 | 27.05 | 89.60 | 52.74 |
| **11.** | **Days to maturity** | **Wild type** | **88.4±0.25** | | | | | |
|  |  | **200 Gy** | 86.23 ± 0.89 | 79.0 - 96.0 | 4.07 | 3.36 | 68.26 | 5.72 |
|  |  | **250 Gy** | 87.12 ± 0.98 | 72.0 - 99.0 | 5.15 | 4.62 | 80.58 | 8.55 |
|  |  | **300 Gy** | 88.15 ± 0.72 | 81.0 - 102.0 | 4.73 | 4.16 | 77.46 | 7.54 |
