# Supplementary material for: Characterization of terminal flowering cowpea (Vigna unguiculata (L.) Walp.) mutants obtained by induced mutagenesis digs out the loss-of-function of phosphatidylethanolamine-binding protein
Source: PLoS One. 2023 Dec 14;18(12):e0295509. doi: 10.1371/journal.pone.0295509 (PMC10721064; doi:10.1371/journal.pone.0295509)
Supplement: S2 Table — (DOCX) [file pone.0295509.s006.docx]

**S2 Table. Variability parameters for 11 morphological traits in M_5_ generation of cowpea mutants.**

| **S. No.** | **Characters** | **GCV** | **PCV** | **ECV** | **Heritablity** | **GA (%) of mean** |
| --- | --- | --- | --- | --- | --- | --- |
| **1.** | **Plant height** | 42.13 | 42.90 | 8.06 | 96.45 | 85.24 |
| **2.** | **Days to flowering** | 30.62 | 31.48 | 7.31 | 94.60 | 61.35 |
| **3.** | **Number of primary branches** | 18.15 | 18.32 | 2.50 | 98.14 | 37.03 |
| **4.** | **Number of clusters per plant** | 18.84 | 20.65 | 8.45 | 83.26 | 35.41 |
| **5.** | **Number of pods per plant** | 21.06 | 22.48 | 7.86 | 88.19 | 40.63 |
| **6.** | **Peduncle length** | 41.96 | 43.11 | 9.90 | 95.61 | 84.13 |
| **7.** | **Pod length** | 43.74 | 44.50 | 8.17 | 97.32 | 88.57 |
| **8.** | **Number of seeds per pod** | 23.94 | 24.57 | 5.51 | 94.97 | 48.071 |
| **9.** | **Hundred seed weight** | 19.86 | 20.26 | 4.01 | 96.07 | 40.104 |
| **10.** | **Days to maturity** | 28.53 | 28.87 | 4.41 | 98.11 | 58.08 |
| **11.** | **Single plant yield** | 38.82 | 39.33 | 6.34 | 97.400 | 78.922 |
