# Supplementary material for: Characterization of terminal flowering cowpea (Vigna unguiculata (L.) Walp.) mutants obtained by induced mutagenesis digs out the loss-of-function of phosphatidylethanolamine-binding protein
Source: PLoS One. 2023 Dec 14;18(12):e0295509. doi: 10.1371/journal.pone.0295509 (PMC10721064; doi:10.1371/journal.pone.0295509)
Supplement: Attachment — Submitted filename: Response to Reviewers.docx [file pone.0295509.s008.docx]

**Response to editor and reviewer comments**

**Editor comments**

Thank you for submitting your manuscript to PLOS ONE. After careful consideration, we feel that it has merit but does not fully meet PLOS ONE’s publication criteria as it currently stands. Therefore, we invite you to submit a revised version of the manuscript that addresses the points raised during the review process. In general, the two reviewers feel the study is technically sound. Please address the minor comments. Please submit your revised manuscript by Sep 28 2023

**Response**

Thank you very much for handling our manuscript and giving us the opportunity to submit a revised draft of our manuscript. We appreciate the time and effort you and the reviewers dedicated to providing valuable feedback on our manuscript. We are grateful to the reviewers for their insightful comments. According to the comments, we have addressed all the issues and revised the manuscript carefully. The changes made in the manuscript are marked with blue color/tracks. We hope the revised version is now suitable for publication We look forward to hearing from you in due course regarding our submission and responding to any further questions and comments you may have.

Thanks for your kind support

**Best wishes**

Prof. Thangaraj Kandasamy

Department of Plant Breeding and Genetics, Agricultural College and Research Institute, Tamil Nadu Agricultural University, Madurai, Tamil Nadu, India.

[ka.thangaraj@gmail.com](mailto:ka.thangaraj@gmail.com)

**Reviewer #1:**

**Comments**

The article "Characterization of terminal flowering cowpea (*Vigna unguiculata* (L.) Walp.) mutants obtained by induced mutagenesis digs out the loss-of-function of phosphatidylethanolamine-binding protein" presents an interesting study on the development and characterization of determinate habit mutants with terminal flowering (TFL) in cowpea. The mutants exhibited desirable traits such as TFL growth habit, longer peduncles, erect pods, increased number of pods per cluster, and early maturity. A significant finding of the study is the identification of a single nucleotide transversion in the *VuTFL1* gene of the determinate mutants resulting in a loss-of-function mutation. and the MutMap population. The identification and characterization of the loss-of-function mutation in the PEBP gene contribute to our understanding of the underlying molecular mechanisms controlling these traits. The article is generally well-written and organized, presenting the methodology, results, and implications of the study in a clear and concise manner. The article will make a valuable contribution to the scientific literature in the field of crop genetics and breeding; therefore, I recommend for publication in PlosONE.

**Response:** On behalf of all the authors, I am writing to express our sincere gratitude for your thorough review and invaluable feedback to our manuscript. Your positive evaluation of the work is truly motivating and reassuring. It gives us great pleasure to know that our collaborative efforts have culminated in this positive outcome. We also appreciate the time you have taken for the revision process and your valuable comments.

**Reviewer #2:**

**Comments**

Dear Authors, This is a very interesting study on cowpea. However, I have two comments.

**Response:** Thank you for your thoughtful review of our manuscript. We sincerely appreciate the time and effort you have invested in evaluating our work. Your feedback is invaluable to us as we strive to enhance the quality and impact of our research.

**Comment 1:** Firstly, the take home message is not very clear:

**Response 1:** Thanks for your kind suggestions. We have rephrased our conclusion to ensure that the main findings and implications were presented more effectively. Kindly check the revised manuscript Abstract and discussion sections.

**Comment 2:** Secondly, the quality of the figures are not up to the mark. Many of the figures are blurred and illegible.

**Response 2:** We apologize for the quality issues you identified in the figures. We have carefully reviewed and improved the resolution of all images using the PACE software, as per the journal's recommendation, to ensure standard of the figures.
